# The mechanism of cytoplasmic incompatibility is conserved in *Wolbachia*-infected *Aedes aegypti* mosquitoes deployed for arbovirus control

**DOI:** 10.1371/journal.pbio.3002573

**Published:** 2024-03-28

**Authors:** Rupinder Kaur, Cole J. Meier, Elizabeth A. McGraw, Julian F. Hillyer, Seth R. Bordenstein

**Affiliations:** 1 Pennsylvania State University, Departments of Biology and Entomology, University Park, Pennsylvania, United States of America; 2 Pennsylvania State University, One Health Microbiome Center, Huck Institutes of the Life Sciences, University Park, Pennsylvania, United States of America; 3 Vanderbilt University, Department of Biological Sciences, Nashville, Tennessee, United States of America; 4 Pennsylvania State University, Center for Infectious Disease Dynamics, Huck Institutes of the Life Sciences, University Park, Pennsylvania, United States of America; Cornell University, UNITED STATES

## Abstract

The rising interest and success in deploying inherited microorganisms and cytoplasmic incompatibility (CI) for vector control strategies necessitate an explanation of the CI mechanism. *Wolbachia*-induced CI manifests in the form of embryonic lethality when sperm from *Wolbachia*-bearing testes fertilize eggs from uninfected females. Embryos from infected females however survive to sustain the maternally inherited symbiont. Previously in *Drosophila melanogaster* flies, we demonstrated that CI modifies chromatin integrity in developing sperm to bestow the embryonic lethality. Here, we validate these findings using *w*Mel-transinfected *Aedes aegypti* mosquitoes released to control vector-borne diseases. Once again, the prophage WO CI proteins, CifA and CifB, target male gametic nuclei to modify chromatin integrity via an aberrant histone-to-protamine transition. Cifs are not detected in the embryo, and thus elicit CI via the nucleoprotein modifications established pre-fertilization. The rescue protein CifA in oogenesis localizes to stem cell, nurse cell, and oocyte nuclei, as well as embryonic DNA during embryogenesis. Discovery of the nuclear targeting Cifs and altered histone-to-protamine transition in both *Aedes aegypti* mosquitoes and *D*. *melanogaster* flies affirm the Host Modification Model of CI is conserved across these host species. The study also newly uncovers the cell biology of Cif proteins in the ovaries, CifA localization in the embryos, and an impaired histone-to-protamine transition during spermiogenesis of any mosquito species. Overall, these sperm modification findings may enable future optimization of CI efficacy in vectors or pests that are refractory to *Wolbachia* transinfections.

## Abstract 2

Wolbachia-induced cytoplasmic incompatibility in fruit flies is known to cause embryonic lethality by modifying chromatin integrity in developing sperm. This study reveals an analogous mechanism in the wMel-transinfected Aedes aegypti mosquitoes that are used to control vector-borne diseases.

## Introduction

Symbiotic biocontrol is a multifaceted strategy that uses microorganisms for positive health outcomes [1]. With the potential to save millions of lives when applied at a scale to global diseases, this approach is highly promising. One example is the recent, international success of deploying an intracellular bacterium of the alphaproteobacterial genus *Wolbachia* in mosquitoes to reduce the transmission of various RNA arboviruses to humans. *Wolbachia* express 2 key phenotypes in these control strategies. The first is a virus-blocking trait [2,3] that reduces the replication of RNA viruses such as dengue, Zika, and Chikungunya in the salivary glands of virus-transmitting mosquito females [4–9]. The second is a symbiotic drive system termed cytoplasmic incompatibility (CI) that results in embryonic lethality when *Wolbachia*-infected males mate with uninfected females [10]. The lethality results from catastrophic, paternal defects in early embryogenesis including failure in condensation and segregation of paternal chromosomes, chromatin bridging, shredding of the paternal nuclei, early embryonic arrest, regional mitotic failures, and nuclear fallout [11–18]. Females carrying the same strain of *Wolbachia* rescue this lethality, thereby conferring a strong fitness advantage that spreads *Wolbachia* in natural host populations [19–22]. The World Mosquito Program (WMP) and others leverage both the virus-blocking and CI traits of the *w*Mel strain of *Wolbachia* from *Drosophila melanogaster* by stably transinfecting it into disease-transmitting *Aedes aegypti* mosquitoes that do not naturally carry *Wolbachia* [23,24]. Mass releases of these *w*Mel-carrying mosquitoes lead to the conditional spread of *Wolbachia* via CI when the bacterial frequency exceeds an unstable equilibrium [25]. Indeed, upon successful releases, the WMP demonstrated 40% to 98% reductions in local dengue cases worldwide [26–33].

In transinfected *Ae*. *aegypti* mosquito hosts, *Wolbachia* induce strong CI [23,25,34], unlike in the natural host *D*. *melanogaster* where CI strength varies [35–43]. Variation in CI strength can depend on various factors such as *Wolbachia* levels in testes [44], host age [35–40], temperature [40–43], maternal symbiont densities [45], and relevant to this study, different *Wolbachia* genotypes interacting in their natural versus artificial host systems. For instance, in *D*. *simulans*, *w*Ri induces nearly complete CI, while *w*No and *w*Ha strains induce lower levels of CI [46]. In the parasitoid wasp genus of *Nasonia*, *w*VitA of *N*. *vitripennis* causes incomplete CI in its native host but nearly complete CI in a related sibling host *N*. *giraulti* [47]. Whether different molecular mechanisms of CI operate by the same *Wolbachia* in varied hosts remains unknown and is the subject of this study.

The genetic basis of *w*Mel cytoplasmic incompatibility is simple with 2 CI factor genes, *cifA* and *cifB*, encoded by WO prophage regions in the *w*Mel genome. Dual transgenic expression in *D*. *melanogaster* testes causes CI, and *cifA* expression alone in ovaries rescues CI [14,48,49]. At the cellular level, Cif_*w*Mel_ proteins act pre-fertilization to impair the histone-to-protamine transition during sperm development and establish CI [50]. Notably, neither of the Cif_*w*Mel_ proteins were detected as paternally transferred to the fertilized *Drosophila* embryos. A similar study examined localization of *w*Pip Cif proteins from *Culex pipiens* in the non-native host *D*. *melanogaster*. Using transgenic flies, a foreign CifB_*w*Pip_ variant in mature CI sperm transfers to the embryo upon fertilization and associates with paternal DNA replication stress [51]. However, paternal chromatin modifications established pre-fertilization in testes and in a wild-type host-*Wolbachia* system were not investigated [51].

Here, we asked the question whether or not *w*Mel transinfected into *Aedes aegypti* recapitulates the cell biological evidence in flies, namely for host modifications of nucleoprotein abundance in developing sperm and the lack of paternal CifB transfer to the embryo. This work thus tests if Cif proteins from the same strain of *Wolbachia* act mechanistically equivalent in native fly versus non-native mosquito hosts [52,53]. We used the WMP’s *Ae*. *aegypti* mosquitoes transinfected with *w*Mel for the majority of the experiments and *Ae*. *aegypti* mosquitoes transinfected with the closely related variant *w*MelM [24]. Both strains induce strong CI with 100% identity in the *cifA* and *cifB* gene sequences. We report that results mostly recapitulated our previous findings in the native, *w*Mel-infected *D*. *melanogaster* host [50]. During mosquito spermiogenesis, *w*Mel Cifs impact paternal chromatin organization by modifying the abundance of histone and protamine nucleoproteins. Paternal transfer of Cif proteins to the fertilized mosquito embryos was not observed, indicating the sperm modified before fertilization establish the paternal-effect lethality of embryos. We further report that CifA occurs in oocytes and embryos of *w*Mel-infected female mosquitoes that rescue CI, which is a new observation since CifA was not detectable in *D*. *melanogaster* embryos. We examine these different findings in the discussion. Overall, the CI/rescue drive system at the heart of vector control efforts depends on nuclear targeting Cif proteins in testes and ovaries. Ensuing modification of developing sperm leads to a disrupted histone-to-protamine transition and thus altered sperm chromatin integrity.

## Results

### *w*Mel CifA and CifB proteins in *Ae*. *aegypti* target nuclei of developing sperm

**Fig 1:**
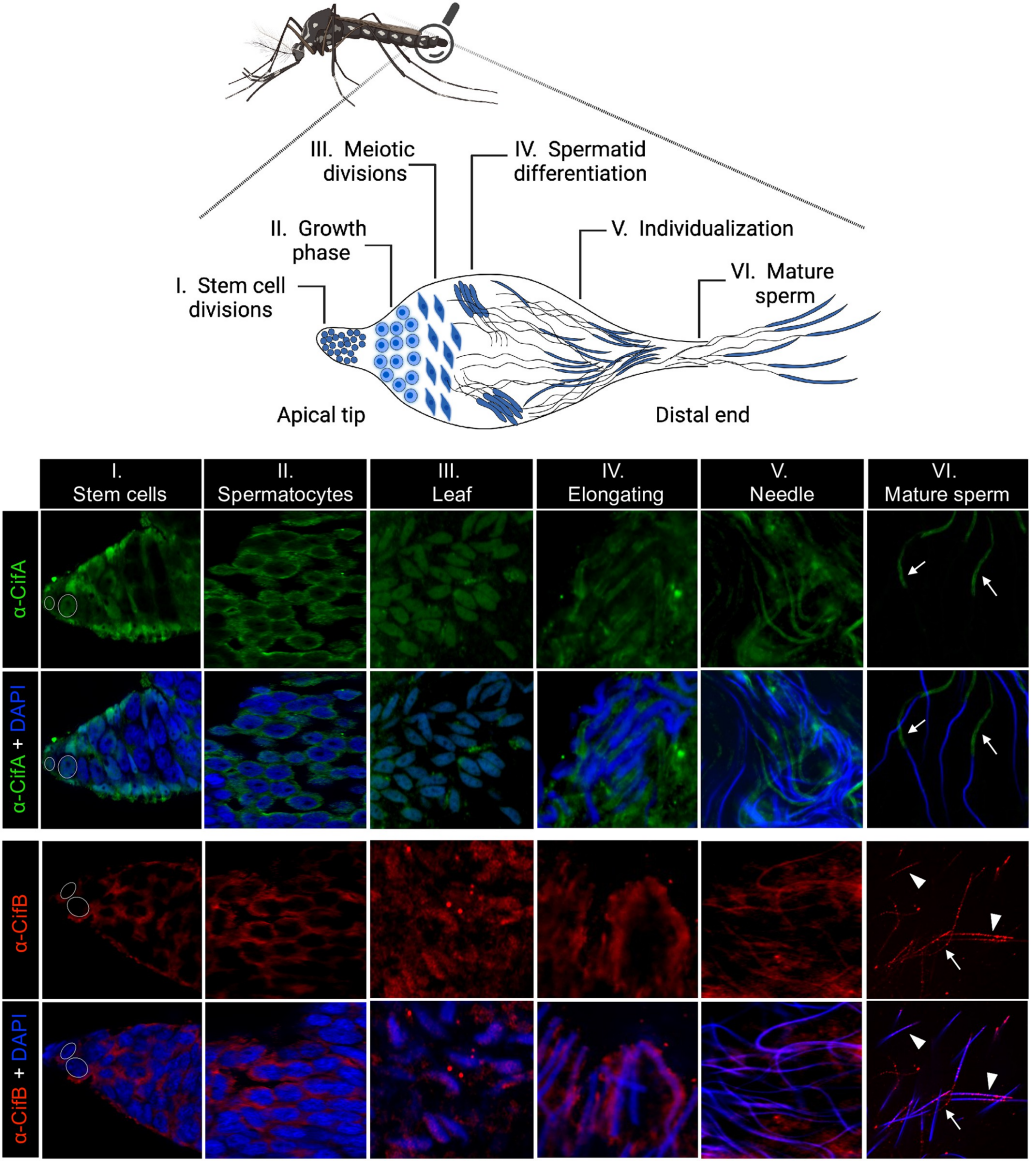
CifA and CifB exhibit cytoplasmic and nuclear localizations during sperm development in *Aedes aegypti*. Schematic representation of *Ae*. *aegypti* male mosquito and magnified reproductive system created with Biorender.com is shown on the top. From the apical end to the base of a testis, cysts progressively increase in size and maturity so that smaller compartments with undifferentiated spermatogonia are found on the apical end, whereas larger cysts containing mature sperm are located at the base or the distal end. *Wolbachia*-infected (*w*Mel+) testes (*n* = 10) from 4 to 5 days old virgin males were dissected and immunostained to visualize CI proteins, CifA (green) and CifB (red), during sperm morphogenesis. DAPI stain (blue) was used to label host nuclei. (I) At the apical end of testes, CifA localizes to germline stem cell nuclei (white circles) with faint signals in the cytoplasm, whereas CifB is strictly in the cytoplasm. (II) Both CifA and CifB localize in the cytoplasm of mitotic spermatocytes in the growth phase and (III) migrate to the nuclei of postmeiotic leaf-stage spermatids, indicating CifA’s cytonuclear exchange behavior. (IV–V) In the later differentiating stages of spermiogenesis, the elongating spermatids harbor CifA surrounding the nuclear periphery, which then strips down along the sperm tail during the tightly compacted, individualized, needle-stage spermatid formation. CifB, on the other hand, decorates the nuclear heads during elongation and penetrates the condensed needle stage nuclei. (VI) Upon full maturation, CifA is at the sperm tail (arrows), and CifB localizes in the form of dense punctae in both mature sperm head (arrowheads) and tail (arrows). Occasionally, CifA’s presence in mature sperm head and not the tail was also detected in 2 out of 10 testes examined, with a similar puncta-based localization pattern of CifB (S1A Fig). CifA and CifB signals are absent in *Wolbachia*-uninfected (*w*Mel−) negative control mosquitoes (S1B Fig). The experiment was repeated twice independently.

To examine the cellular localization of *w*Mel Cif proteins during sperm development, we used our developed Cif_*w*Mel_ antibodies in *Ae*. *aegypti* males [50]. Testes organization in *Ae*. *aegypti* resembles a follicle with different compartments termed cysts (Fig 1) [54,55]. From the apical end to the base of a testis, cysts progressively increase in size and maturity so that smaller compartments with undifferentiated spermatogonia are found on the apical end, whereas larger cysts containing mature sperm are located at the base or the distal end. As maturation proceeds in the testes, spermatogonia grow, proliferate, and differentiate into spermatocytes. Spermatocytes then enter meiosis to generate haploid spermatids, which finally develop into spermatozoa via cytodifferentiation in a process known as spermiogenesis. The dramatic remodeling of the initially leaf-shaped spermatids includes nuclear condensation and elongation forming a sperm head and tail opposite to it. Finally, after individualization, the needle-shaped spermatids develop into mature sperm and release into the seminal vesicle [54,55].

We found that CifA localized in the nuclei of germline stem cells at the apical end of testes, whereas CifB occurred in the surrounding cytoplasm in 4 to 5 days old virgin mosquito males (Fig 1). In spermatocytes, both CifA and CifB were cytoplasmic and then acutely transported to the nuclei of postmeiotic leaf stage spermatids, depicting a cytonuclear transfer during the sperm morphogenesis process. Upon elongation, CifA localized to the cytoplasmic periphery of elongating spermatids, suggesting shuttling of the protein back to the cytoplasm, whereas CifB remained nuclear. After nuclear compaction, CifA stripped down along the tails of needle-shaped spermatids, and CifB remained colocalized with the spermatid head. Mature sperm extracted from the distal end of the testes demonstrated CifA remained with the sperm tail and CifB occurred with both the head and tail of sperm. CifA’s presence in all sperm heads was observed in 2/10 testes examined in one experimental run (S1A Fig). Since *Wolbachia* were not present in the mature sperm, these results specify that Cif proteins are secreted out of bacterial cells to invade and interact with nuclei and cytoplasm of developing sperm cells. In *Wolbachia*-uninfected (*w*Mel-) control testes, no CifA and CifB signals were detected, as expected (S1B Fig).

### *w*Mel hijacks the evolutionary-conserved, histone-to-protamine transition of developing sperm

**Fig 2:**
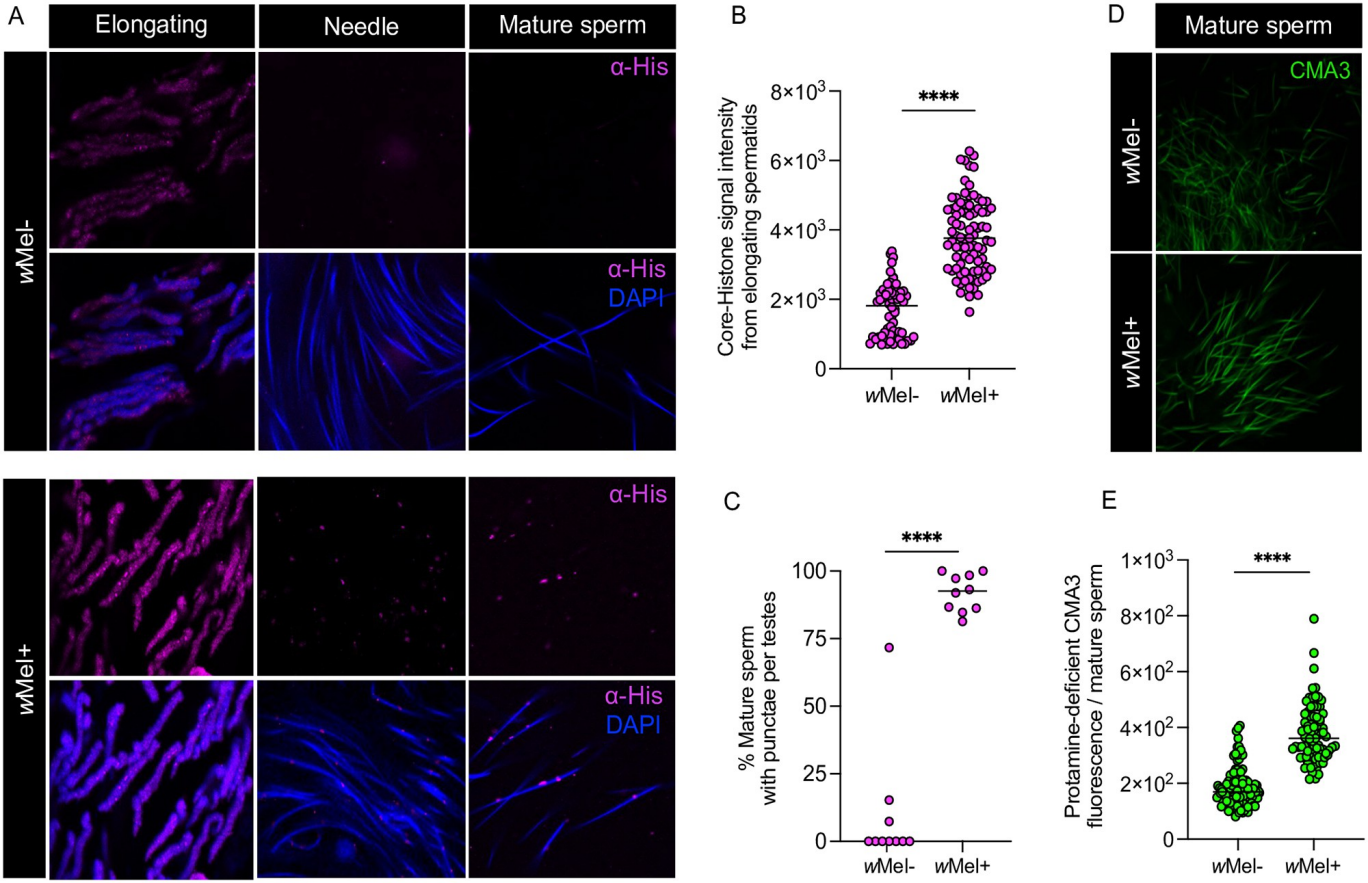
Maturing CI sperm abnormally retain histones and lack protamines. (A) *w*Mel+ and *w*Mel- testes (*n* = 10) from 4 to 5 days old virgin males were dissected and immunostained to visualize and quantify histone abundance (magenta) in elongating spermatids and mature sperm during sperm morphogenesis in *Ae*. *aegypti*. DAPI stain (blue) was used to label spermatid nuclei. (A, B) During the elongating stage, *w*Mel+ males show higher core histones signal intensity than in *w*Mel- males. (A, C) In the late maturation stages, *w*Mel+ males show prolonged histone retention in the form of puncta in condensing needle-stage spermatids and individual mature sperm, whereas histones are removed in *w*Mel- males as expected. A full uncropped image of needle spermatids with retained histones in *w*Mel+ versus *w*Mel- testes is shown in S2 Fig. (D) Fully mature sperm were extracted from *w*Mel+ and *w*Mel- testes (*n* = 10) of 4 to 5 days old virgin males and stained with fluorescent CMA3 (green) for detection of protamine deficiency. Signal intensity in each individual sperm nucleus head was quantified in ImageJ (see Methods) and graphed. *w*Mel+ sperm show enhanced protamine deficiency levels compared to *w*Mel− control. Horizontal bars in (B), (C), and (E) represent median value. Statistical significance (*p* < 0.05) was determined by running pairwise comparisons based on Kolmogorov—Smirnov test. All the *p*-values are reported in S1 Table. The experiments were performed in two independent biological replicates. Raw data underlying this figure can be found in S1 Data file.

Proper sperm chromatin integrity is crucial for successful fertilization and embryogenesis. During spermiogenesis, histone-bound chromatin is changed to a protamine-bound one that tightly condenses sperm DNA to package the sperm head [56]. However, mispackaged sperm due to an abnormal histone-to-protamine transition can cause male infertility and embryonic lethality in diverse systems from insects to mice [57,58]. Thus, to characterize Cifs’ impact on the developing sperm chromatin organization of *Ae*. *aegypti*, we first investigated histone dynamics in infected (*w*Mel+) and uninfected (*w*Mel-) testes using an antibody targeting core histones conserved by sequence and structure across eukaryotes [59,60]. We found 2.01-fold significantly higher core histone intensity in *w*Mel+ elongating spermatids (median = 3,764) compared to *w*Mel- (median = 1,867) (Fig 2A and 2B and S1 Table). The increased histone retention persisted in the form of puncta on the *w*Mel+ needle spermatid (Figs 2A and S2). Upon isolating mature sperm out of the testes, we consistently found a marked and significant increase in the percentage of *w*Mel+ sperm (median = 92.5%) with retained histone puncta compared to *w*Mel- (median = 0%) (Fig 2A and 2C and S1 Table). To detect whether the abnormal histone retention in mature sperm is associated with compounding errors in the histone-to-protamine transition, we used the fluorochrome chromomycin A3 (CMA3) stain that fluoresces upon binding to protamine-deficient regions of DNA [61]. Mature sperm isolated from *w*Mel+ exhibited a similar 2.1-fold significant increase in CMA3 intensity consistent with protamine deficiency (median = 361.1) relative to *w*Mel− (median = 169.7) (Fig 2D and 2E and S1 Table). Notably, this is the first characterization of an impaired histone-to-protamine transition through spermiogenesis in a mosquito species.

### Protamine-deficient sperm travel with Cifs to the female reproductive tract

**Fig 3:**
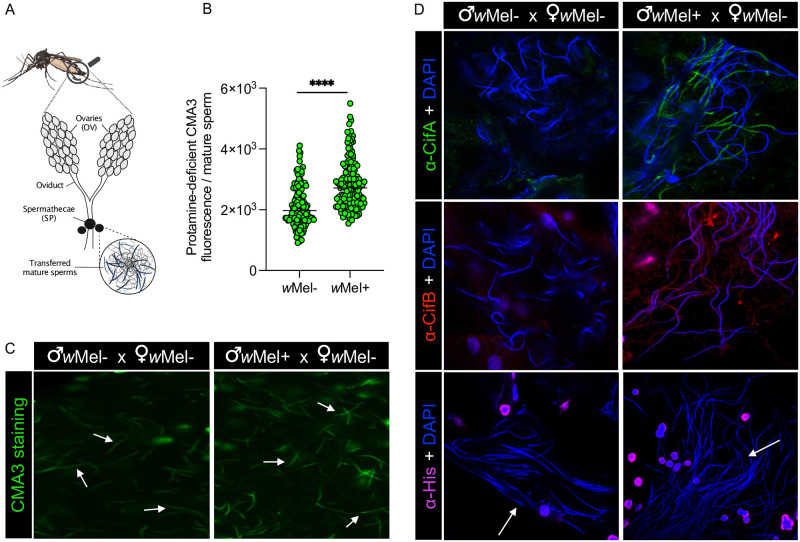
Protamine deficiency and Cifs persist in mature sperm transferred to spermathecae of uninfected females, whereas histones do not. (A) Schematic representation of *Ae*. *aegypti* female reproductive system, created with BioRender.com that shows ovaries (OV) and spermatheca (SP), the specialized sperm storage organ. *w*Mel+ and *w*Mel− males were mated in single pair to *w*Mel− females. Females were then dissected, and transferred sperm were released by puncturing SP. (B) Individual sperm head intensity quantification shows that sperm protamine deficiency from *w*Mel+ males persist after transfer in the females compared to that of *w*Mel- males. Horizontal bars represent median value. Statistical significance (*p* < 0.05) was determined by running pairwise comparisons based on a Kolmogorov—Smirnov test. The *p*-values are reported in S1 Table, and raw data underlying panel B of this figure can be found in S1 Data file. (C) Representative images of CMA3-stained mature sperm (arrows) transferred from *w*Mel- and *w*Mel+ males in *w*Mel- female reproductive systems are shown. (D) Isolated sperm from females were immunostained for localizing CifA (green), CifB (red), and histones (magenta). DAPI stain (blue) was used to label nuclei. In sperm derived from *w*Mel+ males, CifA is predominantly seen along the sperm tail and CifB on both sperm head and tail. Both CifA and CifB signals are absent in sperm from *w*Mel- males. Histone puncta earlier detected in mature sperm from *w*Mel+ testes are not present in sperm extracted from female tract (arrows). All the experiments were performed in 2 independent biological replicates.

To investigate if sperm chromatin modifications and Cif proteins transfer paternally to females after mating, we crossed freshly hatched virgin *w*Mel+ or *w*Mel- males with virgin *w*Mel- females in single pair mating. Individuals mated for 4 days to ensure mating. On day 5, the reproductive tract of females together with the spermathecae (SP)—a set of 3 rigid capsules that store, protect, and nourish sperm [62]—were dissected, and SPs were squashed to disentangle the sperm mass (Fig 3A). CI-sperm derived from *w*Mel+ males transferred to SP of mated *w*Mel- females showed a 1.37-fold significant increase in protamine deficiency (median = 2,717.43) compared to sperm derived from *w*Mel- males (median = 1,974.29) (Fig 3B and 3C and S1 Table). Following Cif antibody staining, we detected CifA on the sperm tail and CifB on the sperm head and tail (Fig 3D), similar to their localization pattern in sperm isolated from testes (Fig 1). Using the histone antibody, we detected no histone signals on sperm isolated from females mated with both *w*Mel+ and *w*Mel- males (Fig 3D), suggesting that histone retention is temporary because they may be lost or degraded before mating or during transfer to the female SP.

### Paternal CifA and CifB are not detected in CI embryos; maternal CifA is present in rescue embryos

Once fertilization occurs, the canonical cellular consequences of CI are delayed paternal chromatin condensation, paternal chromatin bridges, and segregation defects resulting in early, and in some cases, mid-stage embryonic arrest [12–14,17,18,63–67]. In *Ae*. *aegypti*, the male pronucleus is formed within 1 h after fertilization, followed by apposition and 2 successive mitotic divisions producing 4 nuclei between 1 and 2 h [68]. By 3 h, asynchrony of nuclear divisions occurs where mitotic nuclei in various stages of divisions are widely scattered over the deutoplasm, the yolk reserve of the egg.

**Fig 4:**
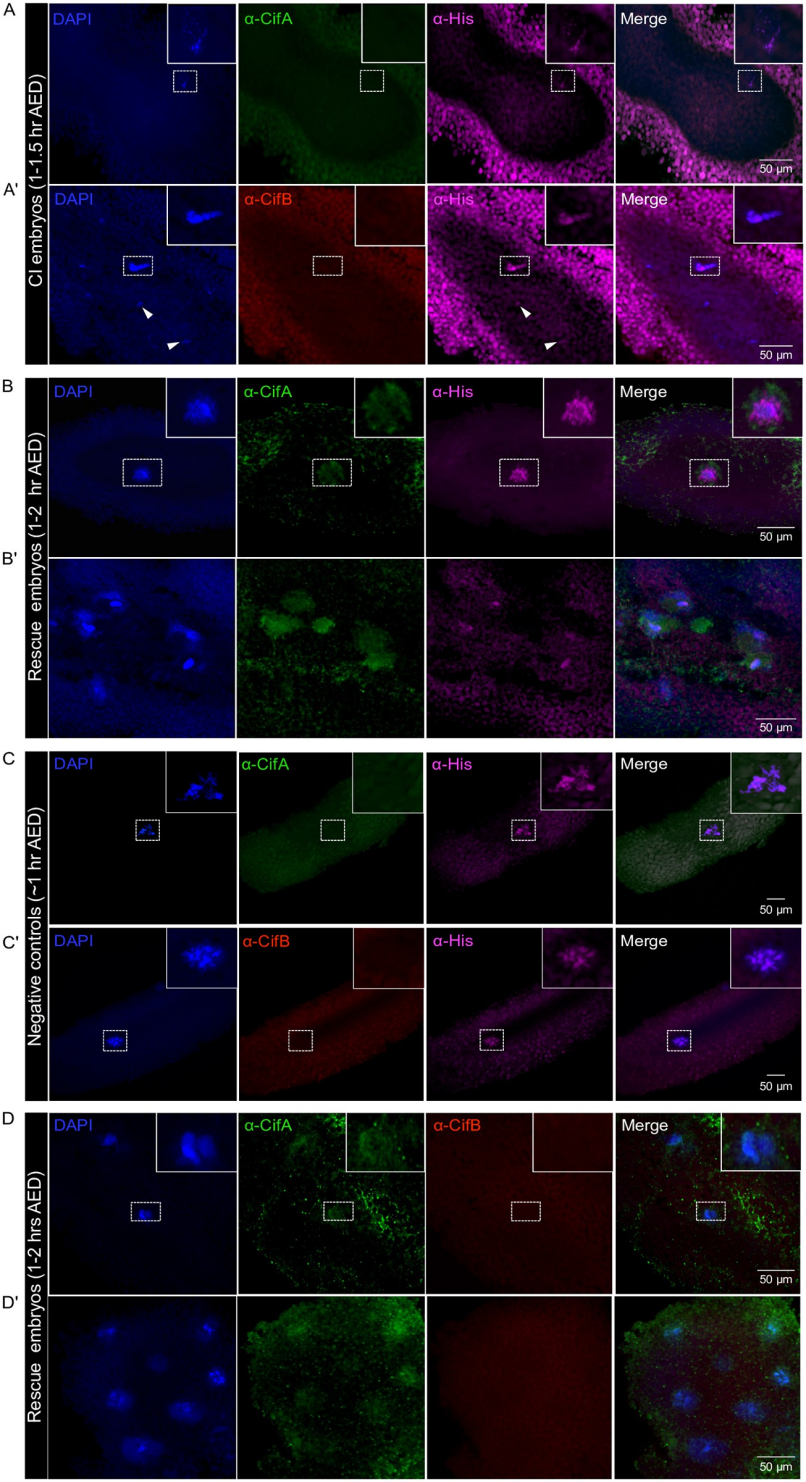
CifA and CifB are not paternally delivered to the *Ae*. *aegypti* CI embryos; CifA is detected in the rescue embryos. Immunofluorescence of CifA (green) and CifB (red) was performed in 1–3 h old, fertilized *Ae*. *aegypti* embryos (*n* = 15) each obtained from CI cross (*w*Mel+ male × *w*Mel− female), rescue cross (*w*Mel+ male × *w*Mel+ female), and negative control cross (*w*Mel- male × *w*Mel- female). Host DNA was labeled with DAPI (blue), and core histone (magenta) was used as a positive antibody control. In 1–1.5 h AED old CI embryos, (A) nuclei fragmentation and (A’) shredding characteristics were observed (arrowheads). Histone signals were detected colocalizing with host DNA, whereas no CifA and CifB signals delivered from paternal sperm were detected. Dotted box is drawn around the chromosomal DNA with magnified view shown in the inset at the upper right corner. (B) In approximately 1 h AED old rescue embryos, CifA localizes in and around the periphery of chromosomal DNA putatively separating at metaphase. (B’) At approximately 2 h AED during nuclear cycle 3, CifA localizes with nuclear DNA and surrounding cytoplasm of 8 daughter cells in rescue embryos (S3 Fig). (C, C’) Approximately 1 h old negative control embryos showed positive histone signals colocalizing with chromosomal DNA and absence of both CifA and CifB, as expected. (D, D’) Further, to investigate whether CifA and CifB could bind in the fertilized rescue embryo, we used both antibodies together (see Methods) and once again validated no transfer of paternal CifB and consistent nuclear and peripheral signals of maternal CifA. We note the autofluorescence due to yolk fat cells and green dots are background noise likely due to the presence of unwashed residual antibody.

To investigate if paternal Cif proteins loaded in the sperm head and tail transfer to the embryos after fertilization, we performed 3 crosses: *w*Mel+ males × *w*Mel- females (CI cross), *w*Mel+ males × *w*Mel+ females (Rescue cross), and *w*Mel- males × *w*Mel- females (negative control). We collected embryos in 0 to 3 h intervals after egg deposition (AED) followed by immunostaining. In approximately 1.5 h old CI embryos, the expected chromatin catastrophes occurred, including fragmented DNA (Fig 4A) and under-condensed nuclear DNA partially scattered in the egg yolk cytoplasm (Fig 4A’). Importantly, neither CifA nor CifB was detected in CI embryos, whereas control histone signals were observed colocalizing with nuclear DNA. These results indicate that Cifs are likely not paternally delivered via sperm to the mosquito embryo, similar to our previous findings in flies [50].

**Fig 5:**
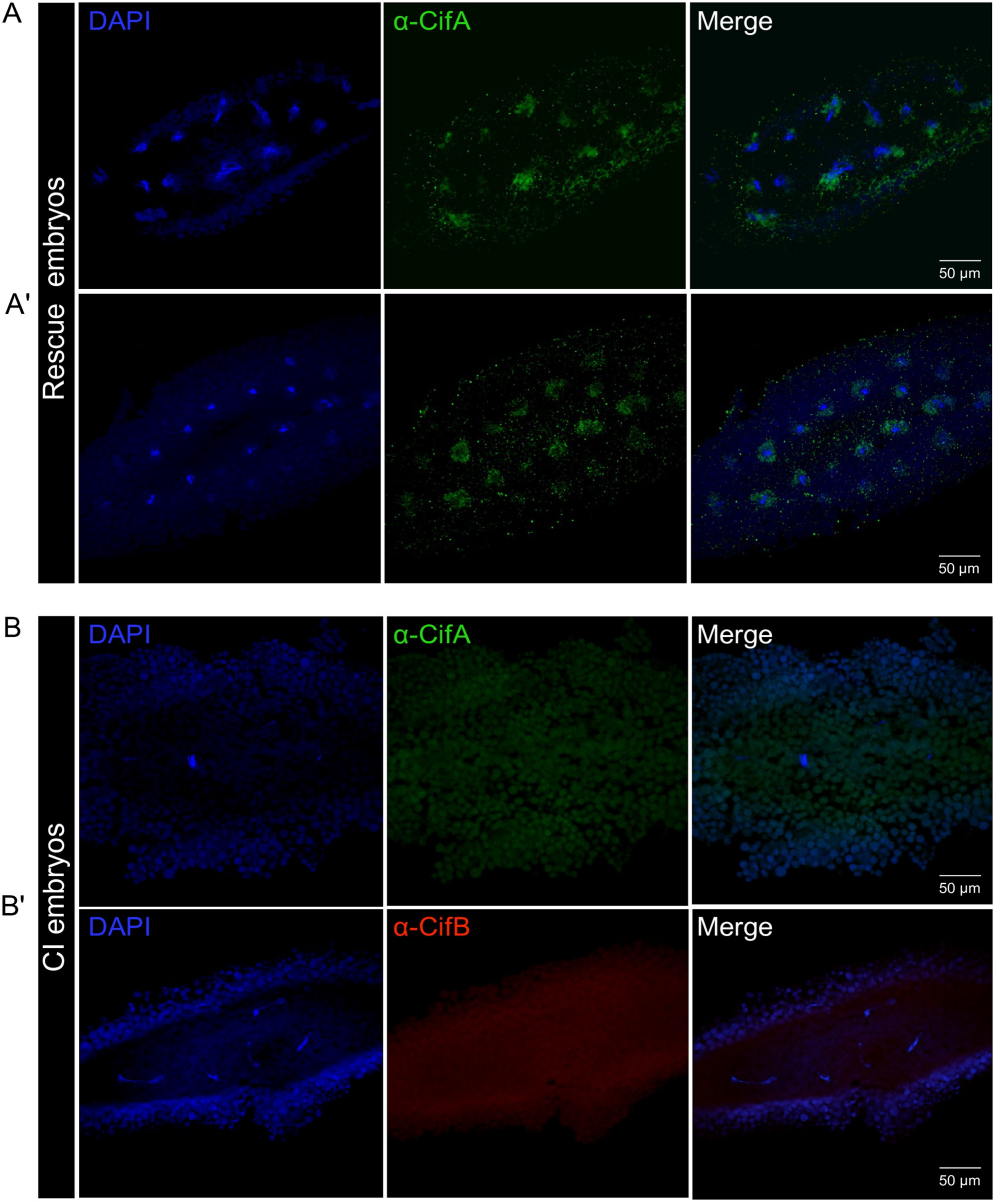
CifA remains attached to the chromosomes and in cytoplasmic periphery around the nuclei in late-stage rescue embryos, whereas CifB is undetected. Late-stage CI and rescue embryos collected at approximately 3 h AED were immunostained with CifA and CifB antibodies. We consistently detected concentrated patchy signals of CifA adjoining the periphery of separating chromosomes at anaphase and in the cytoplasm of divided nuclei at 4 to 5 and later division cycles, respectively (Fig 5A and 5A’). In contrast, the CI embryos collected at the same time showed disrupted nuclear divisions and no signals of neither CifA nor CifB (Fig 5B and 5B’).

In approximately 1 h old rescue embryos, we observed synkaryon that forms after maternal and paternal pronucleus appose each other. Maternally derived CifA partially overlapped with DNA and predominantly concentrated around the periphery of a putative group of chromosomes at metaphase (Fig 4B). The end of the third nuclear cycle in approximately 2 h old rescue embryo resulted in 8 daughter nuclei, where CifA is detected in and around each daughter cell nucleus (Figs 4B’ and S3). In the negative control approximately 1 h old embryos, histone signals colocalized with segregating chromosomal DNA, and no CifA and CifB signals were detected, as expected (Fig 4C and 4C’). We also tested whether CifA and CifB may bind together in early 1 to 2 h old, fertilized, rescue embryos by co-staining with both Cif antibodies (see Methods). While consistent signals of CifA co-localized with DNA, there was no evidence of CifB presence in embryos during initial and subsequent mitotic divisions (Fig 4D and 4D’). In late rescue embryos collected at approximately 3 h AED, we consistently detected concentrated patchy signals of CifA adjoining the periphery of separating chromosomes at anaphase and in the cytoplasm of divided nuclei at 4 to 5 and later division cycles, respectively (Fig 5A and 5A’). In contrast, the CI embryos collected at the same time showed disrupted nuclear divisions and no signals of neither CifA nor CifB (Fig 5B and 5B’), indicating a lack of evidence for paternal Cif transfer to the fertilized embryos.

If Cif expression in these early embryos is regulated by maternally inherited *Wolbachia* cells, then we expect gene expression levels to be high for CifA and low for CifB. qPCR analysis of *cif* gene expression in embryos carrying *w*MelM collected 0 to 2 h AED indeed confirm this expectation with an order of magnitude higher expression for *cifA* (S4 Fig). It remains possible that the CifB protein is not translated due to the low mRNA levels detected. An alternative explanation for the presence of CifA and absence of CifB in early rescue embryos is that CifA is inherited with maternal DNA from the oocyte to the embryo (see below section).

### CifA localizes to the nuclei of germline stem cells, nurse cells, and oocyte during oogenesis in females

**Fig 6:**
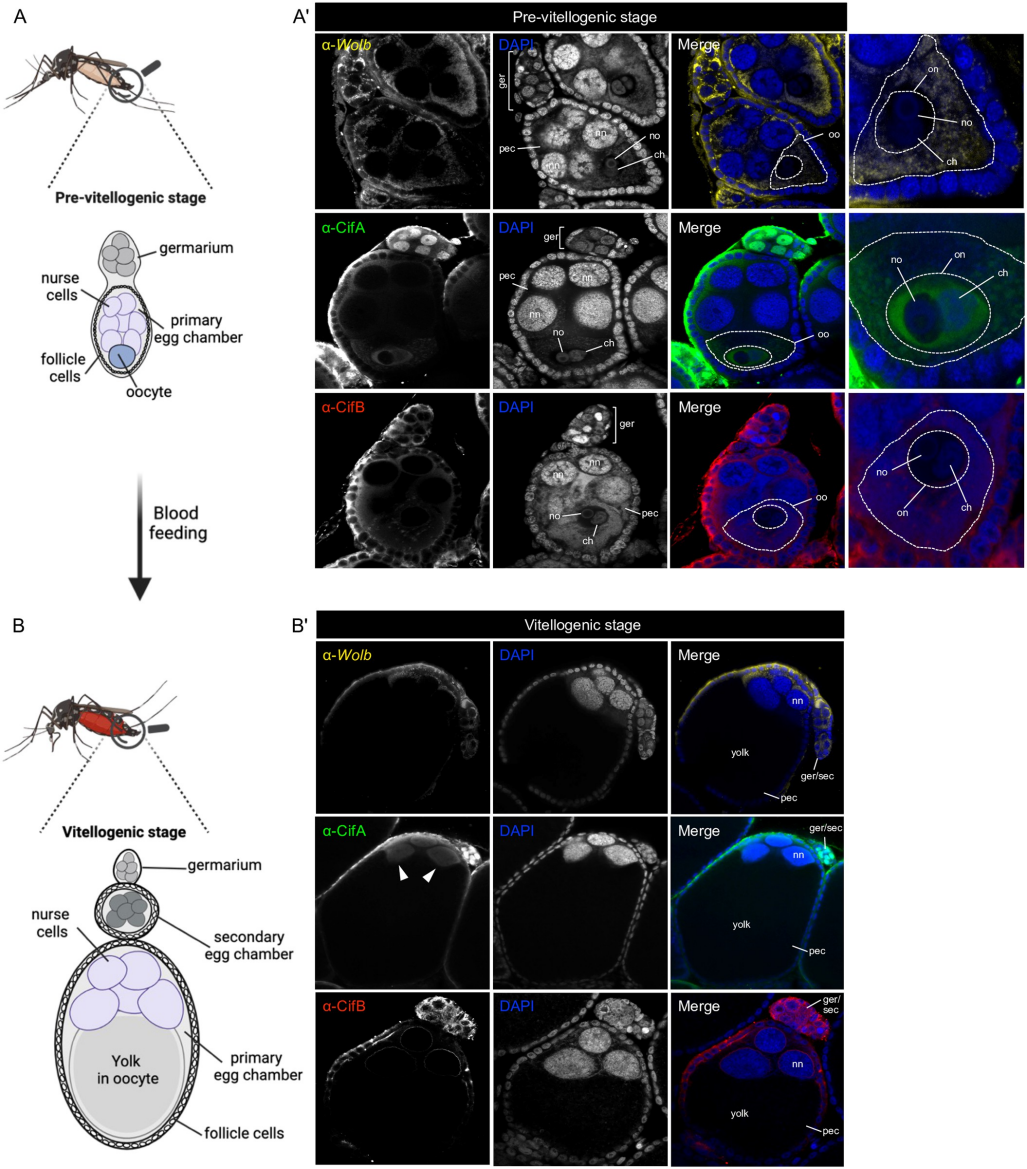
Compared to cytoplasmic *Wolbachia* and CifB in the *Ae*. *aegypti* ovaries, CifA exhibits nuclear localization in germarium, nurse cells and oocyte nuclei. (A, B) Schematic representation of *Ae*. *aegypti* ovary on the left illustrates the stages of oogenesis pre- and post-blood feeding, created with Biorender.com. Ovaries (*n* = 10) from 4 to 5 days old females were dissected before and 24 h after blood feeding. (A’) Immunostaining assay shows that in non-blood fed *w*Mel+ females, *Wolbachia* (yellow) and CifB (in red) localize in the cytoplasm of germarium (ger), nurse cells, and oocyte (oo). CifA (green), on the other hand, is present in the nuclei of germ cells and the peripheral cytoplasm of nurse cells in the primary egg chamber (pec). Occasionally, CifA occurs in nurse cell nuclei (nn) (S4 Fig). In the oocyte, CifA is nuclear (on) inhabiting nucleoplasm space containing chromatin mass (ch) and a nucleolus (no). CifA colocalizes with chromatin outside of the nucleolus area. (B) After blood feeding, *Wolbachia* and CifB in *w*Mel+ females remain cytoplasmic in germarium and at the periphery of nurse cells. CifA remains nuclear in germline stem cells, secondary egg chamber (sec), and nurse cells (arrowheads) in pec. After blood intake, oocyte size grows dramatically filled with yolk (black zone) in the pec. *w*Mel- females before and after blood feeding are devoid of any *Wolbachia* and CifA and CifB signals, as expected (S5 Fig). In the ovaries of both *w*Mel+ and *w*Mel- lines, we note that the occasionally observed autofluorescence in red, green, and yellow channels outlining the tissue morphology does not signify *Wolbachia*, CifA, and CifB signals, respectively.

The transgenic basis of rescue is specifically dependent on *w*Mel CifA expression in fly and mosquito ovaries [48,49,69]. The *in situ* localization dynamics of CifA in mosquito ovaries has not been evaluated to date. Unlike *Drosophila*, oogenesis in *Ae*. *aegypti* occurs in 2 stages (Fig 6A and 6B). The first pre-vitellogenic stage occurs before females feed on blood and specifically affiliates with an increase in primary ovarioles [70]. The second vitellogenic stage starts after blood feeding and associates with deposition of yolk proteins and lipids from the fat body, and RNA and proteins from nurse cells into developing oocytes [71,72]. Oocyte maturation then leads to egg deposition [55,73]. In *Ae*. *aegypti* ovaries, nutritional provisioning via the blood meal is crucial since eggs cannot exogenously source nutrients, and all nutritional requirements for embryogenesis must be packaged during oogenesis [74].

We tracked *w*Mel *Wolbachia* and CifA localization in ovaries before and after blood feeding. In both blood fed and non-blood fed females, *Wolbachia* resided in the cytoplasm of germline stem cells, nurse cells, and the oocyte, as expected (Figs 6 and S5). In contrast, CifA localization was mainly nuclear. Before blood feeding, CifA occurred in stem cell nuclei in the germarium (GSCs) and in the cytoplasm near follicle cells and nurse cells in the primary egg chamber (Fig 6A’). Occasionally, CifA was detected colocalizing with nurse cell nuclei (*n* = 3/10 ovaries examined) (S6 Fig). The *Ae*. *aegypti* mosquito oocyte comprises an uncommon nucleus with 2 distinct bodies—nucleolus and chromosomal mass [75,76]. In the oocyte nucleus, CifA inhabits the nucleoplasm containing the chromatin mass and does not enter in the nucleolus (Figs 6A’ and S6). CifA’s presence in the oocyte nucleus together with the nuclear chromosomal mass suggests its potential interaction with maternal chromatin early in oogenesis, possibly to prime the rescue process. We also tracked CifB localization that, like *Wolbachia*, was found strictly cytoplasmic around germline stem cells, nurse cells, and oocyte nuclei (Fig 6A’).

After blood feeding and as vitellogenesis proceeds, the primary egg chamber increases in size due to an oocyte packed with non-staining yolk particles. CifA persisted in the GSCs and nurse cell nuclei, whereas CifB remained cytoplasmic surrounding the periphery of nurse cell nuclei in primary egg chamber (Fig 6B’). In the large oocyte, we did not detect either of the proteins likely because the antibody did not penetrate through the vitelline membrane in a whole-mount insect tissue preparation [77–79]. Overall, results indicate that CifA targets the nuclei of ovarian germline, nurse cells, and oocyte in a manner that could facilitate host nuclear modification for rescue either earlier in oogenesis or embryogenesis. The finding also corroborated the discovery of CifA’s nuclear localization signal, which is not present in CifB [50].

## Discussion

Large-scale deployments of *Wolbachia*-infected *Ae*. *aegypti* mosquitoes result in population replacement of *Wolbachia*-uninfected mosquitoes with *w*Mel symbiotic individuals, and concordantly, a 40% to 98% reduction in local dengue cases. This relatively new strategy for arboviral disease control is an excellent example of deploying microbial symbionts for positive health outcomes, and it is likely extendable to agriculture health by reducing the burden of crop infectious diseases as well [80]. The main goal of our study was to decipher the cellular and molecular mechanisms of the *w*Mel CI drive system responsible in major part for the population replacement success stories of the WMP. We report below previously unknown aspects of mosquito reproductive biology in relation to symbiotic control: (i) The CI-causing Cif proteins from *w*Mel *Wolbachia* invade nuclei of developing sperm of *Ae*. *aegypti* mosquitoes and cause an abnormal histone-to-protamine transition, a conserved and essential step for normal sperm metamorphosis and genome packaging [56,81]. (ii) Histone—protamine dynamics were obscure in the mosquito literature, and this work provides the first characterization of this transition under the influence of *w*Mel *Wolbachia* in a mosquito host species relevant for human health. (iii) Mature sperm with protamine deficiency are transferred to the female spermatheca (SP) together with Cif proteins, however, neither CifA nor CifB transfer from SP to the uninfected embryos. (iv) In *w*Mel+ ovaries, CifA has nuclear localization, whereas CifB is cytoplasmic before and after blood feeding. (v) Fertilized embryos derived from *w*Mel+ females show CifA localization in the nuclei of early mitotic stage and at the nuclear periphery in the cytoplasm during late division cycles. (vi) Importantly, there is no colocalization or binding of CifA and CifB in the fertilized embryos.

We detected a few differences in *w*Mel Cifs’ localization in the transinfected *Ae*. *aegypti* mosquitoes compared to our previous work shown in the natural host of *D*. *melanogaster* flies [50]. During pre-meiotic spermatogenesis stage, CifB was not detected in the germ cells in *Drosophila*, whereas in *Ae*. *aegypti*, CifB localizes to the germline cytoplasm. Both CifA and CifB are nuclear in fly spermatocytes but cytoplasmic in mosquitoes. During post-meiosis and elongation stages in flies, CifA and CifB located in the presumed acrosomal region of sperm head. In mosquitoes, CifA exits out of the elongating sperm head and localizes to the cytoplasmic periphery, and CifB decorates the sperm nuclei in a distinct puncta manner. Upon maturation, CifA strictly lies on the sperm tail and CifB occurs with the head in dense puncta form and faint signals along the tail. We hypothesize that the differences in localization in flies and mosquitoes might be due to species-specific differences in sperm morphogenesis or chromatin organization and condensation processes [82,83]. Indeed, to detect Cifs in tightly packaged mature sperm, decondensation of mosquito sperm was not necessary as done previously in flies [50], suggesting that mosquitoes have a less condensed sperm architecture than flies even though they possess the same sperm head diameter [84,85]. These differences may underpin the extent and specifics of the *w*Mel protein—chromatin interactions in different host species.

During the postmeiotic elongation process, sperm chromatin undergoes a dramatic reorganization from a nucleosomal histone-based structure to a protamine-based structure. In accordance with our previous data [50], we here showed that the histone-to-protamine transition occurs abnormally in *w*Mel+ *Ae*. *aegypti*. Unlike flies in which retained histones were detected only until elongating late-canoe spermatids [50], punctate histones were detected on the surface of mature mosquito sperm, highlighting once again the species-specificity of *w*Mel protein—chromatin interactions that may be suggestive of variable CI penetrance (fly host, brief histone retention) versus consistently strong CI (mosquito host, long histone retention). We further highlight a 19% higher protamine deficiency levels in mosquitoes that correlates with their high CI levels [23,25,34] compared to flies. The protamine deficiency changes also varied in sperm isolated from females in *w*Mel-*Ae*. *aegypti* versus *w*Mel-*D*. *melanogaster*. While the CI mechanism generally remains conserved in these host species, there might be additional factors at play, such as CI-related variation in sperm size, length, and species-specific sperm DNA compaction [55,83,84] that may affect which sperm successfully fertilize females. Future comparative investigations on sperm genome architecture, relative nucleoprotamine structural organization, and chromosomal arrangement across insect species will be helpful to determine the underlying factors that may explain varying CI penetrance.

After mating, sperm isolated from the female reproductive tract contained CifA along the tail and CifB decorated the head and tail. Cifs were not detected in the fertilized embryo. There are 2 reasons to explain this result. The first is biological, namely that Cifs are shredded off the sperm surface before embryos are fertilized. Indeed, the plasma membrane of mature sperm in mosquitoes and other animals is covered by a thick glycoprotein coat or glycocalyx [86], which upon mating masks the sperm from an immediate female immune attack [87,88]. Within 24 h of storage in the female reproductive tract, *Ae*. *aegypti* sperm shed their glycocalyx to become motile for fertilizing the egg [89]. Thus, it is possible that Cifs are removed during glycocalyx processing or degraded before sperm entry into the egg through exocytosis of acrosomal content [90], as we discussed previously [50]. The second reason can be a technical challenge of detecting Cifs as the proteins may not sufficiently stain in embryos perhaps owing to their low abundance. However, since the Cifs are detectable in mature sperm at the same abundance expected inside eggs, this explanation seems unlikely. Moreover, CifA is detectable in rescue embryos in this study, thus demonstrating that the staining technique works in embryos. Future research may involve experiments with various staining techniques.

Maintaining sperm genomic integrity is important not only for successful fertilization and embryo development, but also to ensure proper transmission of genetic information across generations. During spermatogenesis, replacement of histones by protamines is facilitated by various posttranslational modifications (PTMs). This transition is also the most vulnerable phase of spermatogenesis for the induction of DNA breaks followed by an immediate repair process for regulating chromatin compaction [81,91]. With insufficient PTMs and DNA repair, developing sperm accumulate errors in the epigenome that can transmit to the egg with important implications for the fate of developing embryo [92–94]. Once fertilization occurs, the transferred sperm with an imbalanced protamine content could suffer from remodeling errors in the chromatin structure, leading to DNA breaks that eventually cause chromosome-type aberrations [95]. The repair of sperm DNA breaks is a key factor to ensure proper embryo development, especially when paternal DNA integrity is compromised [96–98]. In mice, zygotes recognize and respond to DNA damage derived from sperm by specifically delaying paternal DNA replication, causing defective embryonic development and ultimately developmental arrest [99]. Indeed, protamine removal is unaffected in CI embryos, and it is the paternal DNA replication that is delayed in CI sperm, with retention of proliferating cell nuclear antigen, a replication stress marker [51,100].

**Fig 7:**
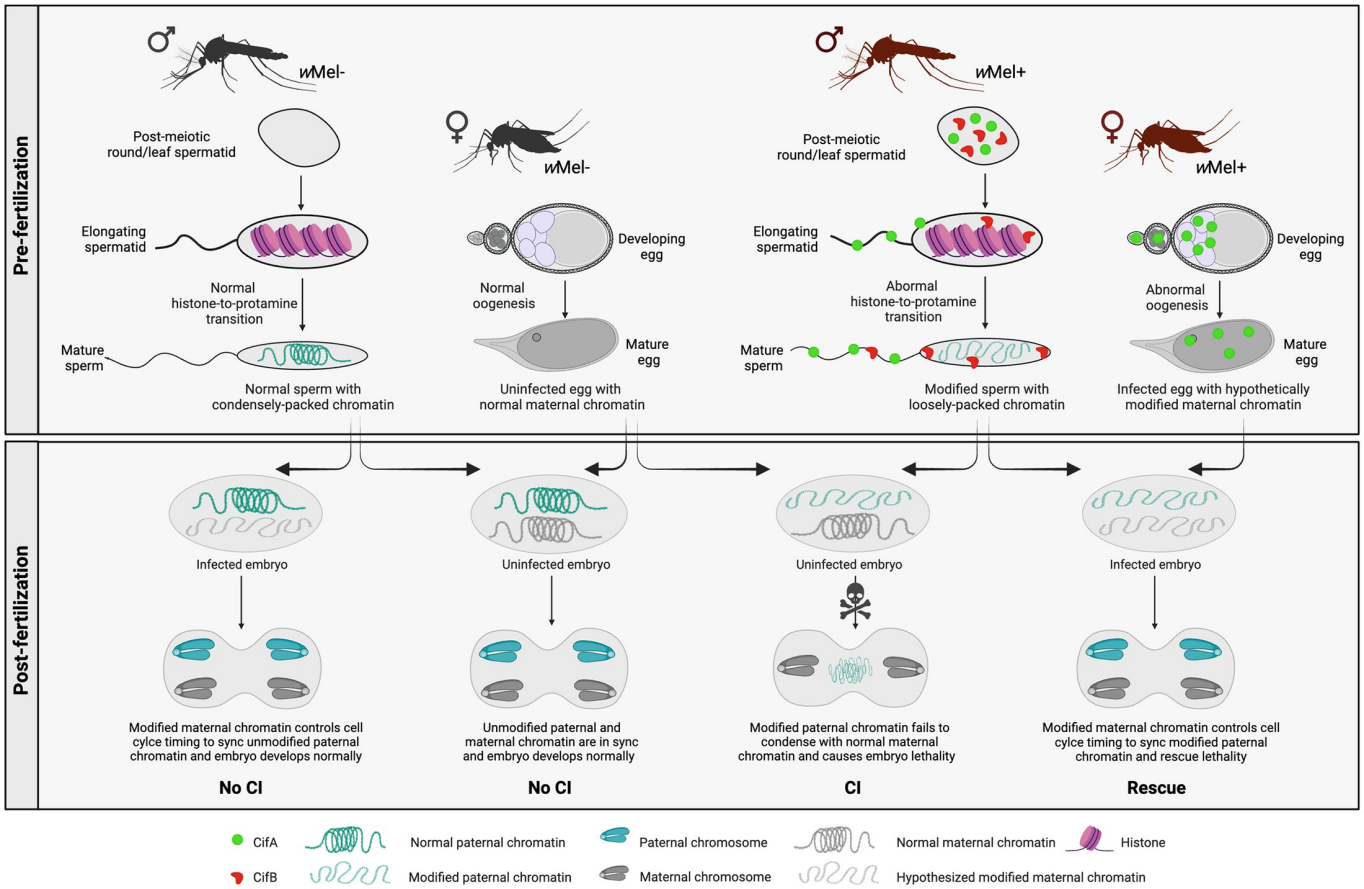
Summary of Host Modification Model of cytoplasmic incompatibility. In *w*Mel-infected *Aedes aegypti* mosquitoes (*w*Mel+), CifA and CifB proteins localize in postmeiotic spermatid nuclei. Upon elongation and maturation, while CifB resides in the sperm nucleus, CifA exits and localize to sperm tail. During elongation, histones wrapped around spermatid DNA are normally removed and replaced by protamines that bind and tightly package the sperm chromatin for maintaining compact genome integrity. In uninfected mosquitoes (*w*Mel-), histone-to-protamine transition occur normally, whereas in *w*Mel+ males, Cif proteins cause abnormal retention of histones and lack of protamine binding to the DNA. Sperm chromatin integrity altered pre-fertilization results in post-fertilization embryonic catastrophe. Upon fertilizing an uninfected egg, the modified sperm chromatin fails to condense and synchronize with maternal chromatin during early mitotic divisions, causing CI-defining embryonic lethality. However, the unmodified sperm from *w*Mel- males will be compatible with embryos derived from either modified (*w*Mel+) or unmodified (*w*Mel-) females because maternal chromatin decides the rate of nuclear mitotic progression. Image was created with Biorender.com.

Thus, we summarize our cell biological knowledge of CI in light of the Host Modification model (Fig 7), in which *w*Mel Cif proteins in males localize and interact with the developing sperm nucleus. Resultantly, the CI sperm develop with compromised RNA and DNA content [101] and irregular histone retention and protamine deficiency in *D. melanogaster* [50] and *Ae*. *aegypti* mosquitoes, observed in this study. A disrupted replacement of histones may alter the epigenetic memory of CI sperm that can be inherited to dictate the fate of embryonic development [102,103]. We speculate that post-fertilization, the modified CI sperm with imbalanced protamine content experience chromatin remodeling defects leading to improper condensation and replication errors. These errors eventually cause embryonic developmental arrest and lethality unless rescued by potentially similar epigenetic changes imparted by *w*Mel+ females expressing CifA during oogenesis and/or embryogenesis as discussed below. The unmodified sperm from *w*Mel- males will be compatible with embryos derived from either modified (*w*Mel+) or unmodified (*w*Mel-) females because maternal chromatin decides the rate of nuclear mitotic progression [104].

*w*Mel CifA in *Ae*. *aegypti* females invade nuclei of germline stem cells, nurse cells, and the oocyte nucleoplasm containing the chromatin mass. These findings are strongly consistent with the nuclear-targeting CifA harboring a functional nuclear localization signal previously reported in fly experiments [50]. Moreover, CifA is biochemically characterized as a RNase [101], and *Wolbachia* is known to cause oxidative stress in *Ae*. *aegypti* ovaries [105]. Therefore, it remains plausible if not likely that *w*Mel CifA in the oocyte nucleus interacts with maternal chromatin and induces changes to ultimately sync with *w*Mel-modified paternal chromatin to rescue CI (Fig 7). Indeed, in mammals, the oocyte is primed to repair genomic integrity of the fertilizing sperm by encoding transcripts that are more abundant in the early embryo than in the later stages [106,107].

After fertilization, no Cif proteins were detected in CI embryos, establishing that paternally encoded, wild-type Cifs are unlikely to transfer to the embryo. It is the fatal paternal genome modification that is necessary to be delivered and manifest CI. Strikingly, we found CifA presence in mosquito embryos derived from *Wolbachia*-infected females where CifA overlaps with nuclei during early mitotic divisions and concentrates in and around the nuclear periphery and in the cytoplasm during later division cycles. While the lack of paternal *w*Mel transgenic and wild-type CifA and CifB transfer is a replicated result across flies and mosquitoes, the presence of CifA in mosquito embryos varies. CifA in flies was only detectable until early oogenesis events; it was absent in embryos [50]. However, in mosquitoes, it is present and affiliated with DNA during early mitotic events of embryogenesis. We speculate that CifA’s presence in the oocyte nucleus of mosquito ovaries (and absence in fly oocytes) explains CifA transfer to mosquito embryos. In association with this difference, we also note that the symbiont density, distribution, and expression of *Wolbachia* in new, transinfected hosts can change dramatically, impacting protein expression, localization, and host phenotypes. Indeed, *w*Mel in *Ae*. *aegypti* is stable and has a higher within-host density [34]. Whether CifA in mosquito embryos is maternally provisioned or embryonically expressed by *Wolbachia* cells requires further investigation. Differential labeling of male versus female pronuclei with specific markers [108] may potentially establish CifA localization to the female pronucleus if it is maternally derived, or to both pronuclei if *Wolbachia*-derived.

Based on *D*. *melanogaster* data, we proposed a CifA-mediated Host Modification Model of rescue whereby CifA modifies host factors during oogenesis (a parallel concept to the CI-induced testes modifications) to impart maternal chromatin changes, which then pass down to the zygote for rescue [49,50]. An updated model based on the *Ae*. *aegypti* data reported here is that CifA proteins can access and/or interact with maternal chromatin not only during oogenesis, but also embryogenesis for maternal chromatin modification to persist and consistently rescue strong CI in this system. Indeed, in murine models, the zygote can repair compromised sperm DNA integrity using maternal DNA repair machinery [109]. However, we caution that it is difficult to test the proximal time and location of the rescue mechanism in a wild-type system where *Wolbachia* are present in both the ovaries and embryos. Transgenic *cif* expression under the control of ovarian- or embryonic-specific expression could, in theory, tease these alternatives apart. In this light, it is noteworthy that transgenic rescue is achieved with diverse CifA variants only by ovarian expression (not embryonic expression) till date [14,49,69,110–112].

Finally, the collective data on *w*Mel CI cellular biology [50] (and this study) in support of Host Modification (HM) model of CI contrasts with that of *w*Pip from *Culex pipiens* mosquitoes studied in a non-native *D*. *melanogaster* host [51]. Our *in vivo* evidence in both flies and mosquitoes establishes that the CI-defining host modifications incipiently launch pre-fertilization, and fatal paternal genome modification is delivered to the embryo to manifest CI. CifA_*w*Mel_ and CifB_*w*Mel_ in the sperm very likely do not transfer to the embryos for inducing CI, and CifA_*w*Mel_ and CifB_*w*Mel_ do not bind in the embryos for rescue. On the other hand, another study [51] claims that the transfer of CifB_*w*Pip_ toxin to the fertilized embryo associated with paternal genome replication stress kills embryos. We highlight that the interpretation of this result, while extendable to the premise that CifB causes paternal genome replication errors in the embryo, has an alternative and more parsimonious explanation with the data here. Namely, the integrity of paternal DNA in the embryo is compromised pre-fertilization, which secondarily results in post-fertilization replication stress. This hypothesis was not tested in the study [51]. Additionally, the study predicts that upon fertilization, the maternal CifA_*w*Pip_ should be present in the egg cytoplasm to bind paternally delivered CifB_*w*Pip_ and neutralize its toxicity for rescuing CI. There is no cell biological evidence for this fundamental premise of the toxin-antitoxin model [51]. Notably, the host genotype can regulate induction and/or suppression of CI and rescue [38,113–115]. Therefore, discrepancies in Cif protein transfer and localization could result from a transgenic artifact. For instance, overexpressed, transgenic CifB_*w*Pip_ in a foreign fly host is likely to act aberrantly from wild-type CifB, the latter of which was not measured in embryos from the natural host *C*. *pipiens*. Here, using the same *w*Mel strain from native *D*. *melanogaster* in non-native *Ae*. *aegypti* hosts, we establish that the mechanism of CI in WMP mosquitoes is largely controlled by the *Wolbachia* strain because histone retention, protamine deficiency, and lack of paternal CifA and/or CifB detection in the embryos are shared facets of CI across both hosts.

CI is the symbiotic drive used by the WMP to rapidly spread *Wolbachia* and replace the wild uninfected *Ae*. *aegypti* population to reduce their vectorial competence and improve global health. While this work characterizes the long-sought mechanism of CI and its conservation across *D*. *melanogaster* and *Ae*. *aegypti*, it also raises the potential for leveraging the altered sperm nucleoprotein composition, if needed, to genetically engineer and optimize CI in vectors or pests that are either refractory to *Wolbachia* infection or lack the capacity for *Wolbachia*-induced CI.

## Materials and methods

### Mosquito strains

*Aedes aegypti* mosquito lines with and without *w*Mel *Wolbachia* infection were obtained from Monash University (*w*Mel) [23] for the majority of the work and subsequently from the University of Melbourne, Australia (*w*MelM) [24] for the qPCR-based *cif* expression experiments in embryos (S4 Fig). Both strains induce strong CI with 100% identity in the *cifA* and *cifB* gene sequences [24]. The colonies were reared and maintained at Vanderbilt University and Penn State University, USA, in environmental chambers at 27 °C and 75% relative humidity on a 12-h light/dark cycle. Eggs were hatched in water and larvae fed a mixture of Koi food and baker’s yeast. Pupae from the *w*Mel+ and *w*Mel- colonies were transferred to six-well plates, and upon eclosion, adults were anesthetized on ice and separated by sex; this procedure prevented mating. Adults were housed in 900 cm3 cages with screen walls and fed 10% sucrose ad libitum. The sexes were maintained separately for experimental purposes.

### Immunofluorescence: Testes

Adult virgin mosquito males (4 to 5 days old) with and without *w*Mel were collected for testes dissection in ice-cold 1× PBS solution. Dissected tissues were fixed in 4% formaldehyde diluted in 1× PBS for 30 min at room temperature and washed in PBS-T (1× PBS + 0.3% TritonX-100) 3 times for 5 min each. Tissues were then blocked in 1% BSA in PBS-T (1× PBS + 0.1% TritonX-100) for 1 h at room temperature. They were then incubated independently with primary antibodies: anti-rabbit CifA (1:500), anti-rabbit CifB (1:500), and anti-mouse Histone (1:1,000) (Millipore Sigma, Cat# MABE71) overnight at 4 °C rotating. After washing in PBS-T 3 times for 5 min each at room temperature, tissues were incubated with secondary antibodies: goat anti-rabbit Alexa Fluor 594 secondary antibody (Fisher Scientific, Cat# A11037) and goat anti-mouse Alexa Fluor 488 (Thermo Fisher Scientific, Cat# A11034) at 1:1,000 dilution for 4 h at room temperature in the dark. Tissues were then washed 3 times for 5 min each in PBS-T and mounted in Vectashield mounting media containing DAPI (Vector laboratories, Cat# H120010). Imaging was performed under Zeiss LSM 880 confocal microscope under constant exposure settings.

### Sperm isolation and Chromomycin A3 staining

Sperm from seminal vesicles of 4 to 5 days old virgin males with and without *w*Mel were extracted on a microscope slide using forceps and fixed in 3:1 vol/vol methanol:acetic acid at 4 °C for 20 min. Excess solution was then removed, and the slide was air-dried. Each slide was treated in the dark for 20 min with 0.25 mg/ml of CMA3 in McIlvain’s buffer (pH 7.0), with 10 mM MgCl_2_. Sperm was then washed in 1× PBS, mounted, and imaged using Zeiss LSM 880 confocal microscope. All images were acquired with the same parameters for each line and did not undergo significant alteration. Fluorescence quantification was performed by scoring fluorescent pixels in arbitrary units (A.U.) within individual sperm heads using ImageJ software as per the details described in [116]. Specifically, an area of interest was manually drawn around individual sperm head in both CMA3 and DAPI channels. “Analyze” function was used to measure the area from DAPI channel and raw integrated density (RawIntDen) from CMA3 channel. RawIntDens is the total signal intensity of all the pixels with that particular area that measured fluorescence CMA3 intensity per sperm head. A corrected total cell fluorescence (CTCF) was then calculated by dividing RawIntDens to the area and graphed. The overlapping sperm areas were omitted from the quantification analysis. Statistical significance (*p* < 0.05) was determined by a pairwise comparison using Mann—Whitney test in GraphPad Prism 10.

### Immunofluorescence: Ovaries

Virgin females (4 to 5 days old) with no blood-feeding and 24 h post blood-feeding with and without *w*Mel were dissected to isolate ovaries. Mosquitoes were fed defibrinated sheep blood for 2 h using a Hemotek artificial membrane feeding system heated to 37 °C (Hemotek, Lancashire, UK). Ovaries were dissected in 1× PBS on ice and processed as previously [50]. Tissues were incubated with primary antibodies α-FtsZ (1:150) (a kind gift from Dr. Irene Newton, Indiana University) to stain *Wolbachia*, α-CifA, and α-CifB (1:500) at 4 °C overnight followed by secondary antibody (Alexa Fluor 594, 1:1,000) for 4 h at room temperature in the dark. Samples were then rinsed and blocked again before incubating at 4 °C overnight. After washing in 1× PBS-T 3 times, samples were incubated with a secondary antibody (Alexa Fluor 594) for 4 h in the dark. Tissues were then washed 3 times for 5 min each in 1× PBS and mounted in Vectashield mounting media containing DAPI (Vector laboratories, Cat# H120010). Imaging was performed with a Zeiss LSM 880 confocal microscope.

### Immunofluorescence: Embryos

Embryos with and without *w*Mel were collected 0 to 3 h AED on moist filter paper substrate to capture first and subsequent mitotic division events [68]. To slow down embryogenesis at preselected times, the moist filter paper was briefly held at 4 °C. The embryo bleaching and dechorionation protocol was developed from [117]. Briefly, the exochorion was removed by incubating embryos in 50% bleach for 40 s followed by thorough rinsing with distilled water to remove all traces of bleach. The embryos were then transferred to fixative solution (1:1 4% paraformaldehyde and heptane) in a microcentrifuge tube and incubated in a 60 °C water bath for 30 min. Fixative was removed and replaced with heptane pre-chilled at −80 °C. Embryos were incubated at −80 °C for 5 min. After adding room temperature methanol, embryos vials were vigorously shaken under warm running water for 30 s. The top heptane layer and bottom methanol layer was removed, and embryos were washed 3 to 4 times with fresh methanol to remove residual heptane. Fixed embryos were stored at −20 °C until further use.

Before proceeding to the immunofluorescence assay, the endochorion was manually peeled according to previously established methods [118]. Peeled embryos were then rehydrated with 1× PBS for 5 min followed by 2 times 10 min rinses in 1× PBS-T. Samples were then blocked in 1% BSA in PBS-T for 1 h at room temperature. For samples incubated with 2 primary antibodies generated in different host species, all staining and imaging steps were carried out as for testes and ovary tissues detailed above. For the double-immunolabeling experiments in which the 2 primary antibodies were generated in the same host species (α-CifA and α-CifB in rabbit), we followed previously established protocols [119,120]. Briefly, samples were first incubated with the α-CifA antibody for 1 h at room temperature, rinsed 3 times for 5 min in PBS, and incubated overnight at 4 °C with a DyLight 488-conjugated goat Fab’ fragment anti-rabbit IgG (Jackson ImmunoResearch Laboratories, West Grove, PE, USA) at a 1:100 dilution in PBS. Samples were then rinsed 6 times for 5 min in PBS, incubated with α-CifB for 1 h at room temperature, rinsed 3 times for 5 min in PBS, and then incubated with a goat anti-rabbit Alexa Fluor 594 secondary antibody for 4 h in the dark at room temperature. Samples were then washed 3 times for 5 min each in 1× PBS and mounted in Vectashield mounting media containing DAPI (Vector laboratories, Cat# H120010). Imaging was performed under Zeiss LSM 880 confocal microscope using constant exposure settings.

### RNA isolation and qPCR: Embryos

Mosquito embryos with *w*MelM were collected 0 to 2 h AED on moist filter paper substrate for RNA isolation. The collected samples were immediately manually crushed with RNA-free pestles (USA Scientific, #1415–5390) in liquid nitrogen. RNA was extracted using the Direct-zol RNA MiniPrep Kit (Zymo) followed by DNase treatment (Ambion, Life Technologies), and 1 μg of total RNA was used to synthesize cDNA with SuperScript VILO kit (Invitrogen, #11755250) and cDNA was diluted 1:10 using Ambion nuclease-free water for use in qPCR. qPCR was performed on a QuantStudio 6 Pro Real-Time PCR system using PowerTrack SYBR Green Mastermix (Applied Biosystems, #A46109) with the following conditions: 50 °C 2 min, 95 °C 10 min, 40× (95 °C 15 s, 60 °C 30 s), 95 °C 15 s, 60 °C 1 min, 95 °C 15 s. *cifA* and *cifB* primers and *Wolbachia*-specific *groEL* gene primers were used as described earlier [14,49]. Each reaction was run in 2 technical replicates. Expressions of *cifA* and *cifB* relative to *groEL* were compared using the 2^-ΔCt^ method. Ct values above 30 were omitted from the analysis.

## Supporting information

S1 Fig CifA occasionally localize to mature sperm in *w*Mel+ males. Cifs are absent in *w*Mel- testes. Testes (*n* = 10) from 4 to 5 days old males of wild-type *w*Mel+ and *w*Mel- *Ae*. *aegypti* were dissected and immunostained to visualize CifA (green) and CifB (red) during sperm morphogenesis. DAPI stain (blue) labeled nuclei. (A) CifA was localized in puncta form to the mature sperm head in 1 out of 10 *w*Mel+ testes examined. (B) Both CifA and CifB signals are absent in *w*Mel- control mosquito testes. The experiment was conducted in parallel to the one shown in Fig 1.
(TIF)

S2 Fig Needle spermatids in *w*Mel+ males abnormally retain histones relative to *w*Mel- ones. *w*Mel+ and *w*Mel- testes (*n* = 10) from 4 to 5 days old virgin males were dissected and immunostained to visualize and quantify core-histone abundance (magenta) in needle spermatids in *Ae*. *aegypti*. DAPI stain (blue) labeled spermatid nuclei. *w*Mel+ males show prolonged histone retention in the form of puncta in condensing needle-stage spermatids, whereas histones are removed in *w*Mel- males as expected. Solid arrow indicates somatic histone signals forming testes with no differentiating signals in *w*Mel+ and *w*Mel- testes, as expected. The dotted arrow shows histones specific to developing sperm under *w*Mel+ infection, which are the subject of this study. The images are related to needle spermatid data shown in Fig 2.
(TIF)

S3 Fig CifA localizes in and around the nuclei in rescue embryos. Related to Fig 4, CifA localizes with nuclear DNA and surrounding cytoplasm of 8 daughter cells in rescue embryos collected at approximately 2 h AED during nuclear cycle 3. Upon higher exposure of DAPI channel in gray, the top panel shows there is an embedded nucleus (indicated by white arrow) of another daughter cell that surfaces in a different z-plane as shown in the bottom panel.
(TIF)

S4 Fig *cifA* gene expression is approximately 10-fold higher than *cifB* in early *Ae*. *aegypti* embryos. Embryos from *w*MelM *Wolbachia* strain aged 0–2 h AED were tested for gene expression analysis of *cifA* and *cifB* relative to *Wolbachia groEL* gene. Horizontal bars represent median value. Statistical significance (*p* < 0.05) was determined by running pairwise comparisons based on Mann—Whitney U test. All the *p*-values are reported in S1 Table. Raw data underlying this figure can be found in S1 Data file.
(TIF)

S5 Fig Cifs and *Wolbachia* are absent in *w*Mel- ovaries. (A, B) Immunostaining assay shows that during both pre- (A) and post- (B) blood feeding, *w*Mel- ovaries are devoid of any *Wolbachia* (red), CifA (green), and CifB (yellow) signals, as expected. The experiment was conducted in parallel to the one shown in Fig 6. We note autofluorescence observed in red, green, and yellow channels outlining the tissue morphology does not signify *Wolbachia*, CifA, and CifB signals, respectively.
(TIF)

S6 Fig CifA occasionally localizes to nurse cell nuclei. Immunostaining assay shows that in non-blood fed *w*Mel+ females, CifA (in green) localize in the nuclei of germarium (ger), nurse cells (arrowheads), and oocyte (oo). Within the oocyte, CifA is present in the oocyte nucleus (on) inhabiting nucleoplasm space containing chromatin mass (ch) and a nucleolus (no). CifA colocalizes with chromatin outside of the nucleolus area.
(TIF)

S1 Table *P*-values associated with all statistical comparisons made for quantification data. (XLSX)

S1 Data Raw metadata underlying figures. (XLSX)

## Abbreviations

AED: after egg deposition
CI: cytoplasmic incompatibility
CTCF: corrected total cell fluorescence
HM: Host Modification
PTM: posttranslational modification
SP: spermathecae
WMP: World Mosquito Program
