# Supplementary figures and images for: The mechanism of cytoplasmic incompatibility is conserved in Wolbachia-infected Aedes aegypti mosquitoes deployed for arbovirus control
Source: PLoS Biol. 2024 Mar 28;22(3):e3002573. doi: 10.1371/journal.pbio.3002573 (PMC11014437; doi:10.1371/journal.pbio.3002573)

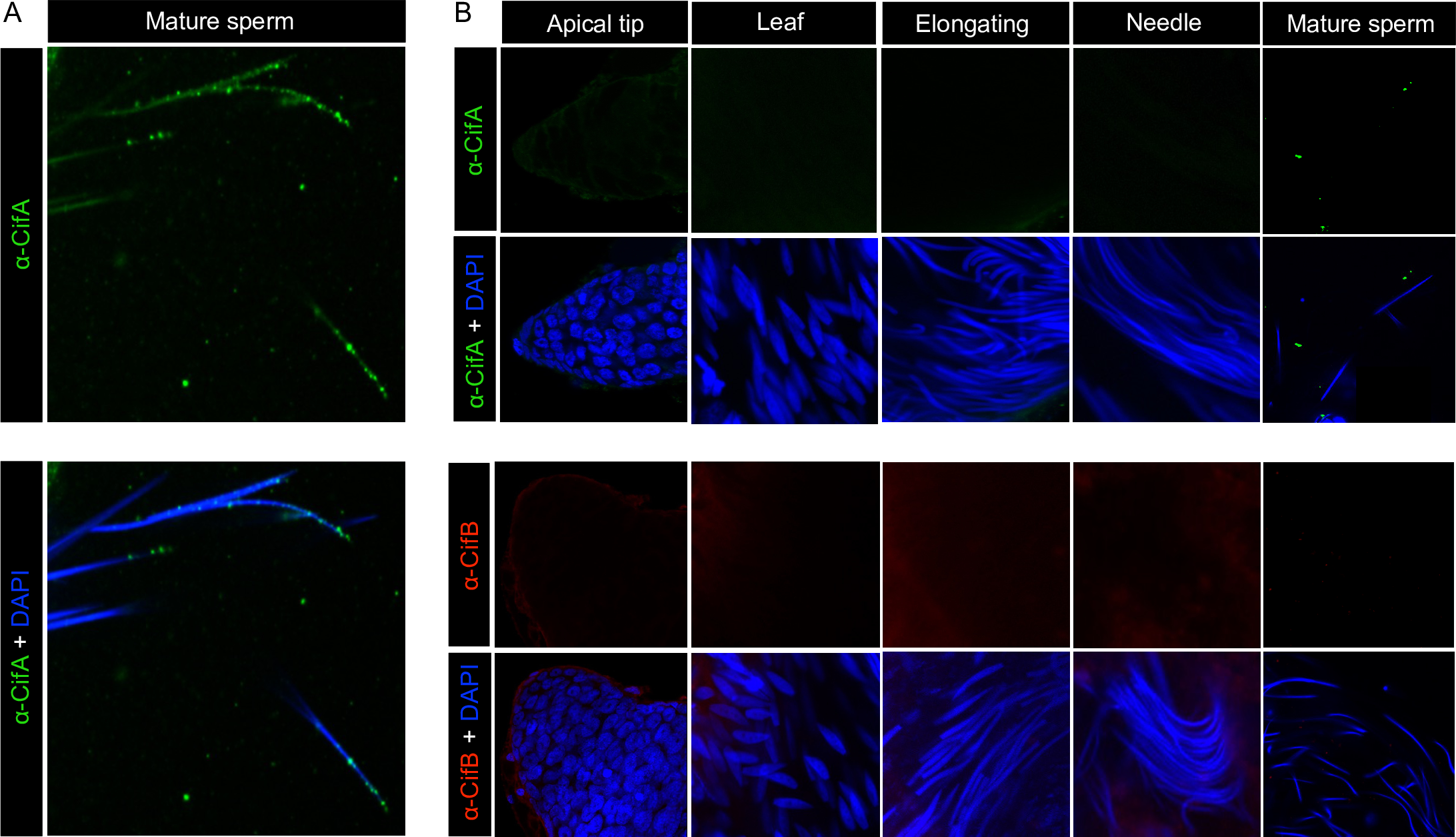

Supplement: S1 Fig — Testes (n = 10) from 4 to 5 days old males of wild-type wMel+ and wMel- Ae. aegypti were dissected and immunostained to visualize CifA (green) and CifB (red) during sperm morphogenesis. DAPI stain (blue) labeled nuclei. (A) CifA was localized in puncta form to the mature sperm head in 1 out of 10 wMel+ testes examined. (B) Both CifA and CifB signals are absent in wMel- control mosquito testes. The experiment was conducted in parallel to the one shown in Fig 1. (TIF) [file pbio.3002573.s001.tif]

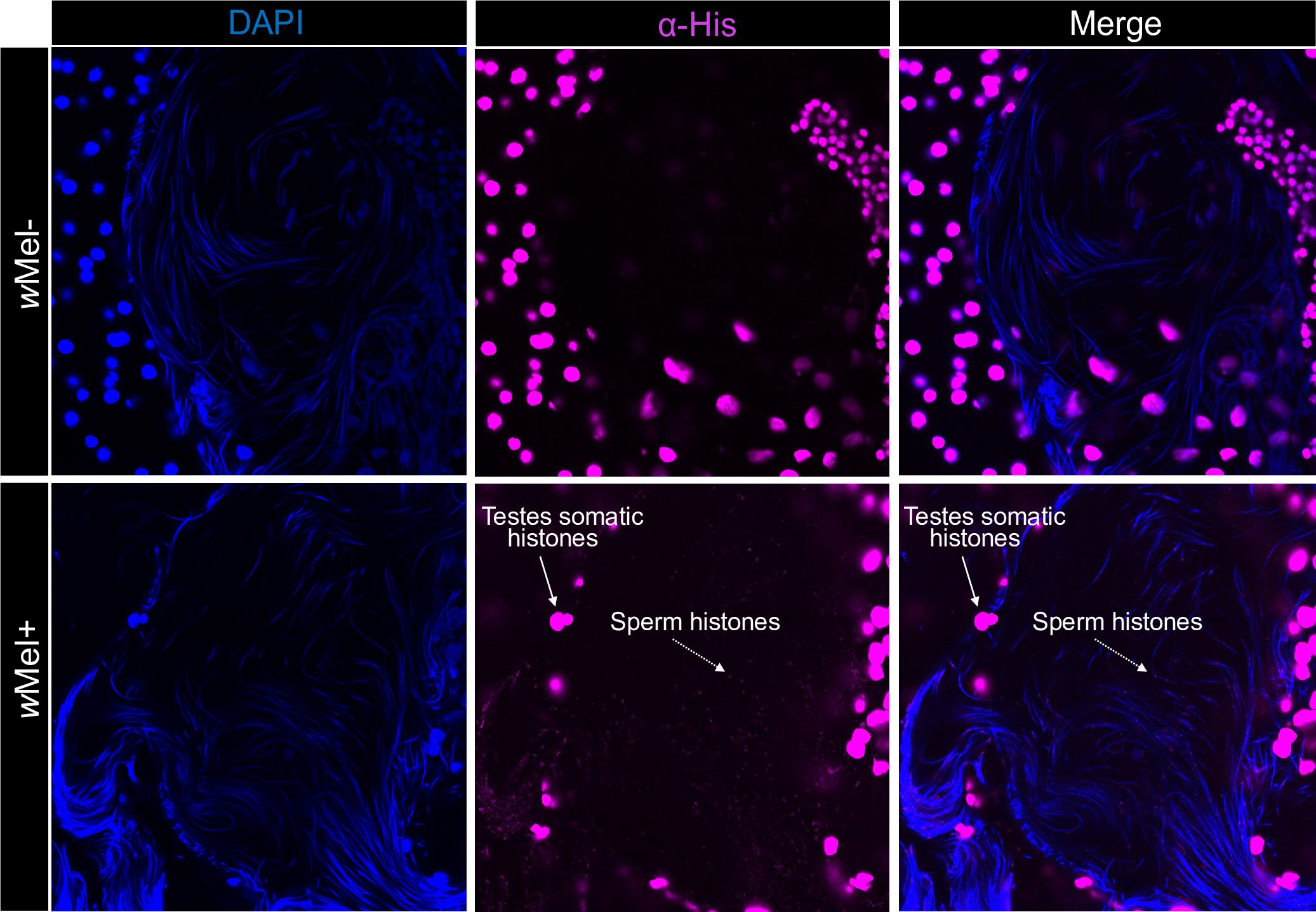

Supplement: S2 Fig — wMel+ and wMel- testes (n = 10) from 4 to 5 days old virgin males were dissected and immunostained to visualize and quantify core-histone abundance (magenta) in needle spermatids in Ae. aegypti. DAPI stain (blue) labeled spermatid nuclei. wMel+ males show prolonged histone retention in the form of puncta in condensing needle-stage spermatids, whereas histones are removed in wMel- males as expected. Solid arrow indicates somatic histone signals forming testes with no differentiating signals in wMel+ and wMel- testes, as expected. The dotted arrow shows histones specific to developing sperm under wMel+ infection, which are the subject of this study. The images are related to needle spermatid data shown in Fig 2. (TIF) [file pbio.3002573.s002.tif]

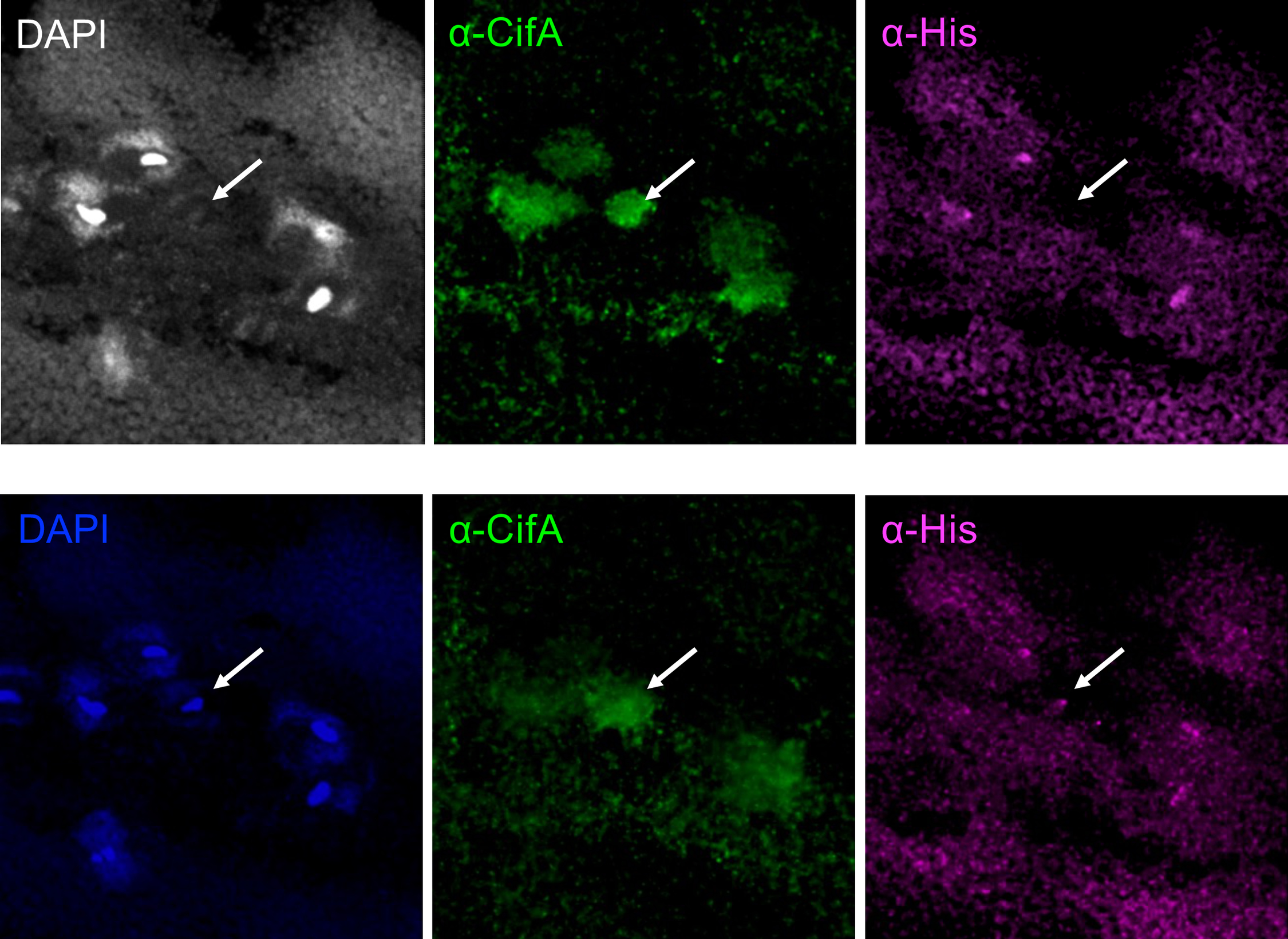

Supplement: S3 Fig — Related to Fig 4, CifA localizes with nuclear DNA and surrounding cytoplasm of 8 daughter cells in rescue embryos collected at approximately 2 h AED during nuclear cycle 3. Upon higher exposure of DAPI channel in gray, the top panel shows there is an embedded nucleus (indicated by white arrow) of another daughter cell that surfaces in a different z-plane as shown in the bottom panel. (TIF) [file pbio.3002573.s003.tif]

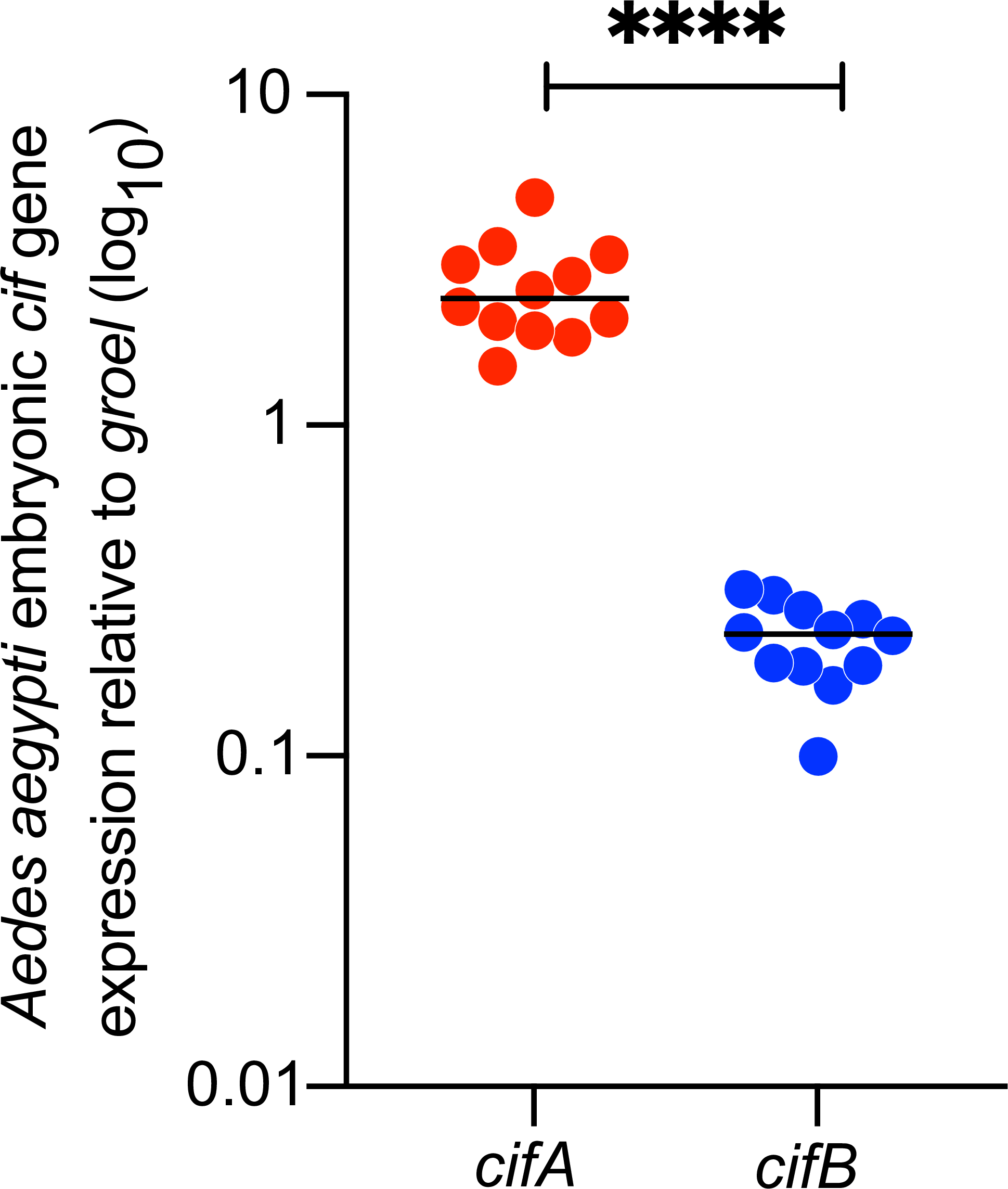

Supplement: S4 Fig — Embryos from wMelM Wolbachia strain aged 0–2 h AED were tested for gene expression analysis of cifA and cifB relative to Wolbachia groEL gene. Horizontal bars represent median value. Statistical significance (p < 0.05) was determined by running pairwise comparisons based on Mann—Whitney U test. All the p-values are reported in S1 Table. Raw data underlying this figure can be found in S1 Data file. (TIF) [file pbio.3002573.s004.tif]

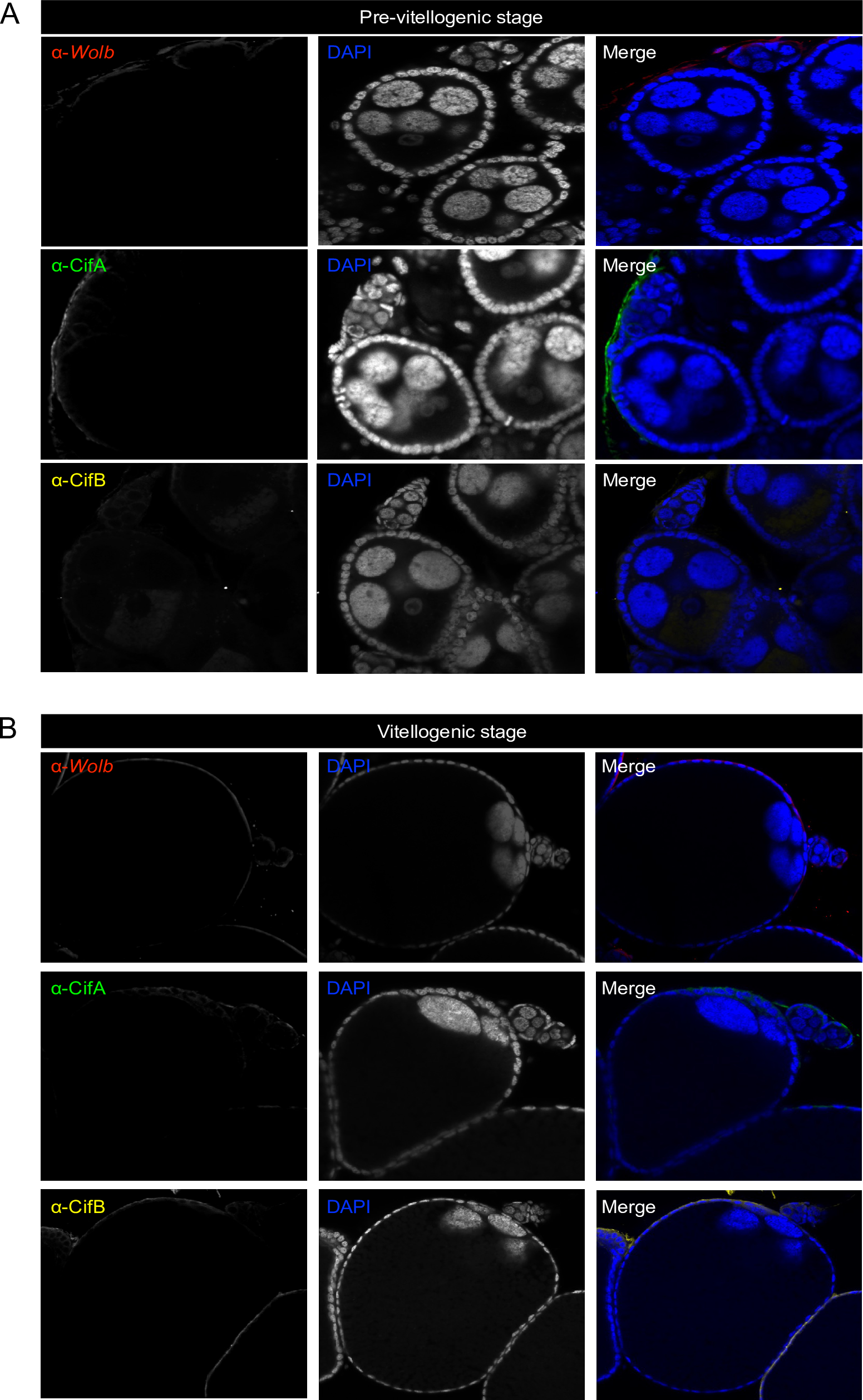

Supplement: S5 Fig — (A, B) Immunostaining assay shows that during both pre- (A) and post- (B) blood feeding, wMel- ovaries are devoid of any Wolbachia (red), CifA (green), and CifB (yellow) signals, as expected. The experiment was conducted in parallel to the one shown in Fig 6. We note autofluorescence observed in red, green, and yellow channels outlining the tissue morphology does not signify Wolbachia, CifA, and CifB signals, respectively. (TIF) [file pbio.3002573.s005.tif]

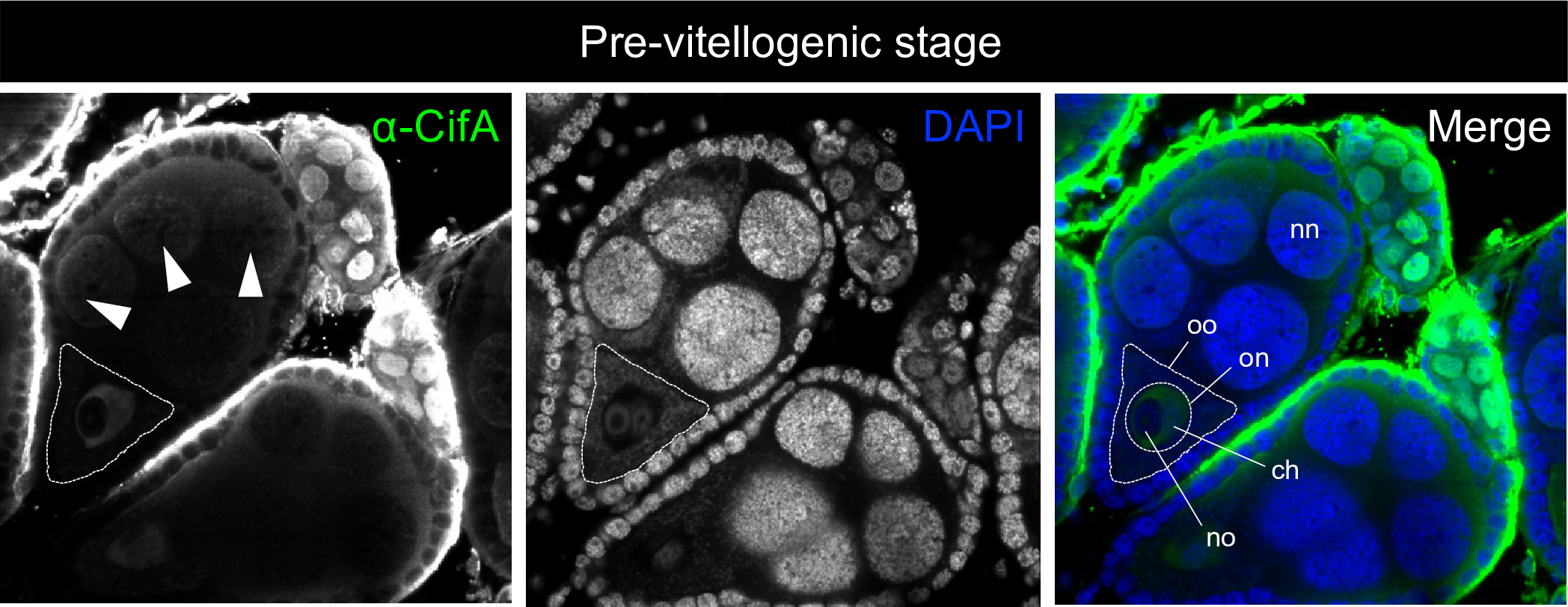

Supplement: S6 Fig — Immunostaining assay shows that in non-blood fed wMel+ females, CifA (in green) localize in the nuclei of germarium (ger), nurse cells (arrowheads), and oocyte (oo). Within the oocyte, CifA is present in the oocyte nucleus (on) inhabiting nucleoplasm space containing chromatin mass (ch) and a nucleolus (no). CifA colocalizes with chromatin outside of the nucleolus area. (TIF) [file pbio.3002573.s006.tif]
